# COVID-19 Smell Impairment and Crosstalk with Hypoxia Physiology

**DOI:** 10.3390/life12091408

**Published:** 2022-09-09

**Authors:** Andrea Mazzatenta, Margherita Maffei, Camillo Di Giulio, Giampiero Neri

**Affiliations:** 1 Neurophysiology, Olfaction and Chemoreception Laboratory, Physiology and Physiopathology Section, Neuroscience, Imaging and Clinical Sciences Department, G. d’Annunzio University of Chieti-Pescara, 66100 Chieti, Italy; 2 Istituto di Neuroscienze, Consiglio Nazionale delle Ricerche, 56124 Pisa, Italy

**Keywords:** COVID-19, smell, hypoxia, pO_2_, pCO_2_, hemoglobin, olfactory threshold, hyposmia, anosmia

## Abstract

Since its apomorphic appearance in 2019, severe acute respiratory syndrome Coronavirus type 2 (SARS-CoV-2) nowadays circulates as a plesiomorphic human virus in several synapomorphic variants. The respiratory tract is the most important site of infection, the viral effects in the lungs are well described, and more than half of the patients could develop shortness of breath and dyspnea and require ventilatory support. The physiological sign of this condition is the decrease in the partial pressure of oxygen in the blood, leading to acute hypoxia, which could be a factor in the disease. In severe patients, we recorded several physiological parameters: breath frequency (BF), partial pressure of oxygen in the blood (pO_2_), partial pressure of carbon dioxide in the blood (pCO_2_), hemoglobin (Hb), heart rate (HR), and blood pressure in correlation with the olfactory threshold. We found significant correlations between reduced olfactory threshold with pO_2_ and hemoglobin levels, changes in heart rate, and increased HR and pCO_2_. These results suggest that COVID-19 causes an impaired sense of smell that decreases in threshold corresponding to the disease severity.

## 1. Introduction

Coronaviridae family members recurrently circulate in the human population and are largely responsible for acute viral nasopharyngitis, mild respiratory disease, and respiratory syndromes, e.g., severe acute respiratory syndrome Coronavirus (SARS-CoV) and the middle east respiratory syndrome Coronavirus (MERS-CoV). In late 2019, a new zoonotic infectious respiratory disease, coronavirus disease 2019 (COVID-19), was reported in China as a novel coronavirus, Coronavirus type 2 (SARS-CoV-2), that was an apomorphic virus [1,2,3,4,5]. Nowadays, the virus circulates as plesiomorphic human infection in several synapomorphic variants.

Viral attachment to the surface of target cells occurs through angiotensin-converting enzyme 2 (ACE2) and transmembrane protease cellular serine (TMPRSS2) [6,7,8], widely expressed in many cell types and organs [9]; it displays an extremely high expression in the characteristic cells of the nasal epithelium, goblet cells, and hair cells [10]. Therefore, these cells are pivotal sites for viral infection and possible reservoirs for its spread. The virus is of the enveloped type that does not require cell lysis for its release. It can exploit the secretory pathways of the nasal goblet cells for continuous, low-level release in the early phase without any discernible illness [10]. Nasal swabs frequently showed higher viral loads than in other areas [10]. Consequently, the nasal epithelium must be considered the initial gateway for the infection and may also act as a reservoir for its spread [11]. The respiratory tract is, therefore, one of the sites of infection and related symptoms are fever, shortness of breath, loss of smell and taste, coughing, and fatigue. COVID-19 ranges from the healthy carrier without symptoms, or with minor symptoms, to the patient with severe progressive pneumonia, up to acute respiratory distress syndrome, coagulation, and multiple organ failure resulting in death [1,3]. High lactate dehydrogenase levels have been found in critical SARS patients and the deceased [11]. Inflammation-related C-reactive protein indicates severe pulmonary infections [12] and decreased hemoglobin [13], but not anemia, are indicators of the disease.

ACE2 is also involved in hypertension, regulating blood pressure through negative modulation of the renin-angiotensin system (RAS), acting as a vasoconstrictor of the ACE–Ang II axis [14]. Neurological consequences are another interesting phenomenon, replicating previous effects observed in MERS-CoV and SARS-CoV [15,16,17,18,19,20,21]. The prominent neurological symptom is the loss or diminishment of the sense of smell and taste [16,22,23,24,25]. According to various studies, the olfactory system from the sensory epithelium, via the olfactory bulb and olfactory nerve, could spread the virus across synapses and trans-synaptic transfer to the central nervous system (CNS) [26]. In the CNS, there are two sets of neuronal networks crucial for respiratory rhythm: the pre-Bötzinger complex (PBC), which is primary in rhythmogenesis, and the retrotrapezoid nucleus/parafacial respiratory group (RTN/pFRG), which is the secondary oscillator [27]. Failure of PBC results in death due to respiratory deficiency [28].

Consequently, chemoreceptive alteration has become a biomarker of infection, even without other symptoms [29]. In the extensive work on SARS-CoV-2, the effects on the lungs are well described; in more than half of the patients, shortness of breath and dyspnea may develop, and more than 10% may require ventilatory support. However, in some papers, a subtle correlation emerges between the neurological effects on the sense of smell and other physiological parameters [2,11]. Among the physiological parameters of COVID-19, the decrease in pO_2_ is crucial; if prolonged, it leads to acute hypoxia, the determining factor in the progression and severity of the disease [11]. The carotid body (CB) plays a role in the modulation of chemoreceptive function and is connected to the renin-angiotensin system [30]. CB type 1 chemoreceptors release dopamine in response to rapid changes in arterial pO_2_, pCO_2_, and pH, thereby increasing sinus nerve activity and provoking centrally mediated cardiopulmonary responses [31]. CB involvement in COVID-19 has been hypothesized because it expresses ACE2 [32,33], behaving similarly to acute mountain sickness hypoxia (AMS) [34].

In infection, weakly related to gender, there is a dichotomy in the severity of the disease, only partially explained by comorbidities, such as hypertension, diabetes, asthma, and renal dysfunction [35]. AMS shows a similar dichotomy in the severity of the disease [36], the high-altitude pulmonary or cerebral oedema leading to sexual dimorphism, with a more significant impact in males [37]. Thus, like COVID-19, some can manage the hypoxic environment, while others fail to acclimatize, and this is not easily explained [38]. Furthermore, the olfactory threshold decreases in lowlanders at high altitudes. It restores slowly when returned to sea level suggesting a link between internal and external chemoreceptors and hypoxia [39].

In the present study, we have investigated olfactory impairment severity, compared with other physiological outcomes of SARS-CoV-2 in severe patients. We looked at a potential link between smell injury and physiological markers of hypoxia as potential crossed biomarkers in the disease and its severity.

## 2. Materials and Methods

The study was conducted following the Declaration of Helsinki and ethical principles of the Belmont Report and approved by the Institutional Review Board local ethic board ‘G. D’Annunzio’s University and Local Sanitary Agency 2, with the number code colf01.2020.

The study evaluated the smell and taste of patients with severe COVID-19 infection in assisted breathing at ‘Ss. Annunziata’ University Hospital COVID-19 special ward. SARS-CoV-2 RNA confirmed the infection by RT-qPCR laboratory testing, days from positive swab range 5–10. Subjects with acute or chronic sinonasal pathologies, who had undergone nasal cavity surgery, recent dental treatments, or with a history of smell or taste disorders, were excluded from the study. All patients were subjected to ENT evaluation, with anterior rhinoscopy and Olfactory test by using a disposable 4-item Olfactory Smart Threshold (OST test Asteria Healthcare for complete method and test–retest reliability refs. [25,40]). The OST test consists of a logarithmic scale of liquid n-butanol to assess by positive answer at a green vial considered normosmia, orange for hyposmia, and red as the severe hyposmia threshold; no answer means anosmia. The odorless white vial is the test’s negative control. The whole procedure takes less than 2 min.

The congruent COVID-19 physiological parameters were recorded: breath frequency (BF), blood oxygen partial pressure (pO_2_), blood carbon dioxide partial pressure (pCO_2_), hemoglobin (Hb), heart rate (HR), and arterial blood pressure BPmax and BPmin, as summarized in [3]. Age classes analysis, according to ref. [40], were collected as follows: 10–19 class n.1; 20–29 n. 2; 30–39 n. 3; 40–49 n. 4; 50–59 n. 5; 60–69 n. 6; 70–79 n. 7; 80–89 n. 8 and 90–99 n. 9. Statistical elaboration MANOVA, nested ANOVA, and one-way ANOVA were used, with the α-level set to 0.05, *p* < 0.05, or noted differently as significant. The commercial software statistical packages were used for all the data and statistical analyses (Statistics 7, StatSoft Inc., Tulsa, Ok, USA; OriginLab Co., Northampton, MA, USA).

## 3. Results

The study evaluated the smell and taste of one hundred patients (mean age 63 ± 15 SD, range 28–94 y.o.; 70 males and 30 females) with severe COVID-19 infection in assisted breathing.

Olfactory threshold impairment has been investigated using an objective quick test, validated, and already tested on COVID-19 patients in [25], with the test–retest reliability at 0.89 by Pearson correlation.

A preliminary MANOVA on physiological parameters returned significant differences *p* < 0.05 for olfactory threshold, sex, and their interaction (F_(7,99)_ = 4.11; F_(7,99)_ = 5.47; F_(7,188)_ = 4.61), the significance of the post-hoc nested ANOVA is *p* < 0.001 (threshold, F_(14,198)_ = 4.52 and threshold × sex, F_(28,396)_ = 8.00).

Olfactory threshold testing results vs. physiological parameters BF, pO_2_, pCO_2_, Hb, HR, BPmax, and BPmin values, in turn, will be presented.

### 3.1. Olfactory Threshold vs. Breath Frequency

**Figure 1:**
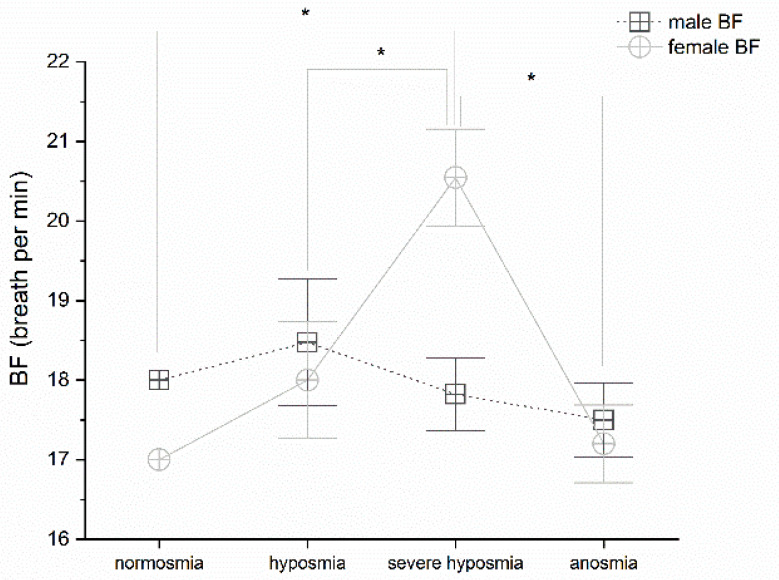
Breath frequency (BF) in male and female vs. olfactory threshold value. Threshold impairment correlate only in female BF, post hoc one-way ANOVAs * *p* < 0.05.

Olfactory threshold detriment correlates with breath frequency variation in female; MANOVA *p* < 0.05, F_(3,25)_ = 7.52 and post-hoc one-way ANOVAs in females show significant differences *p* < 0.05 between normosmia and severe hyposmia, F_(1,14)_ = 11.77; hyposmia and severe hyposmia, F_(1,16)_ = 6.65; severe hyposmia and anosmia, F_(1,15)_ = 11.83, (Figure 1).

### 3.2. Olfactory Threshold vs. pO_2_

**Figure 2:**
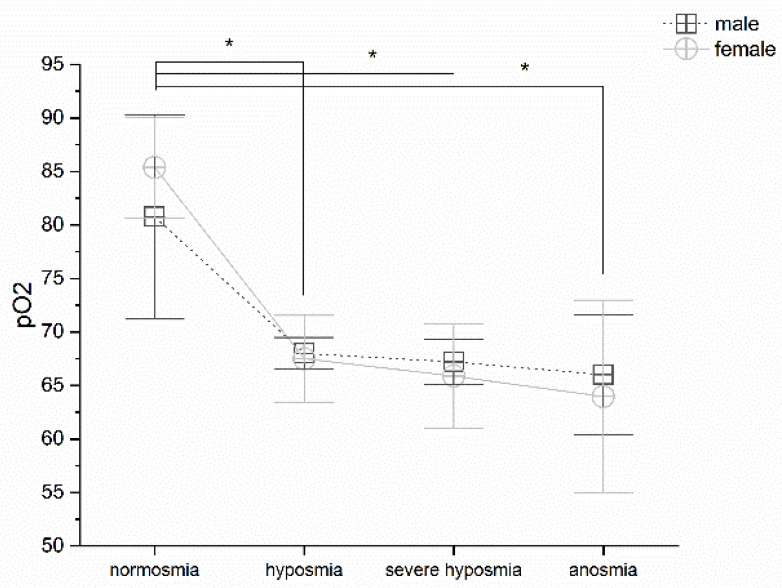
pO_2_ in male and female vs. olfactory threshold value. Threshold impairment correlates with pO_2_ reduction, post hoc one-way ANOVAs * *p* < 0.05.

Olfactory threshold reduction correlates with pO_2_; MANOVA returns significant differences *p* < 0.05, F_(3,99)_ = 3.41. Post-hoc one-way ANOVAs show significant differences *p* < 0.05 between normosmia vs. hyposmia, severe hyposmia and anosmia (F_(1,32)_ = 20.29; F_(1,58)_ = 8.03 and F_(1,18)_ = 6.26); no significant differences occur between hyposmia, severe hyposmia, and anosmia, (Figure 2). No differences occur in gender.

### 3.3. Olfactory Threshold vs. pCO_2_

**Figure 3:**
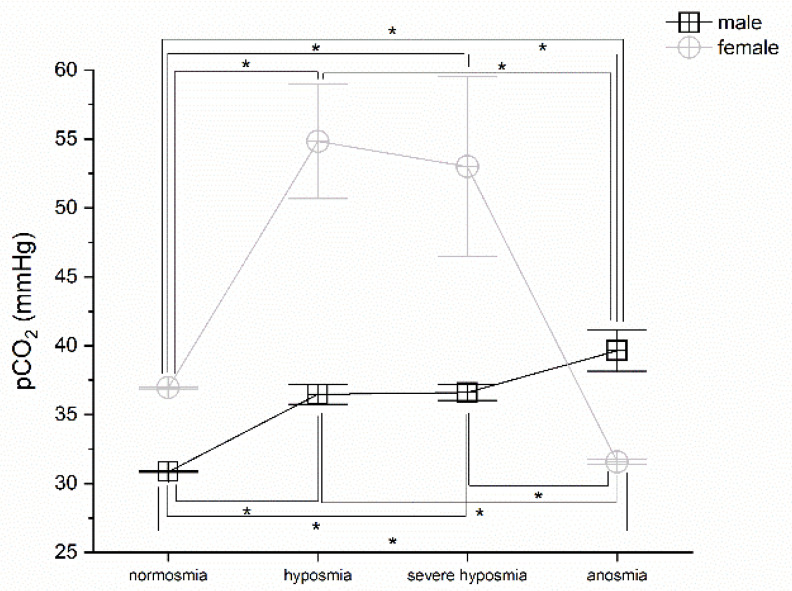
pCO_2_ vs. olfactory threshold value in males and females. Threshold impairment correlates with pCO_2_, post hoc one-way ANOVAs * *p* < 0.05.

Olfactory threshold impairment correlates with pCO_2_; MANOVA returns significant differences in both males and females *p* < 0.05, F_(3,72)_ = 3.66 and F_(3,25)_ = 3.34, respectively. Post hoc one-way ANOVAs show significant differences *p* < 0.05 between normosmia vs. hyposmia, severe hyposmia, and anosmia in males (F_(1,22)_ = 5.75; F_(1,43)_ = 4.92; F_(1,9)_ = 7.89), between hyposmia and anosmia, F_(1,28)_ = 4.70, and severe hyposmia and anosmia female, F_(1,49)_ = 4.51. In females, *p* < 0.05 between normosmia vs. hyposmia and anosmia (F_(1,9)_ = 11.86; F_(1,8)_ = 661.6), between hyposmia and anosmia, F_(1,10)_ = 25.62, and severe hyposmia and anosmia, F_(1,15)_ = 4.75 (Figure 3).

### 3.4. Olfactory Threshold vs. Hemoglobin Levels

**Figure 4:**
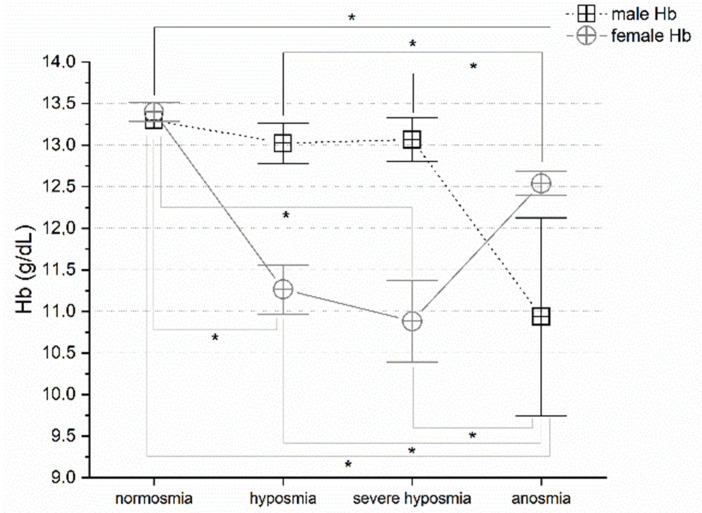
Hb concentration vs. olfactory threshold in males and females. In both sexes, threshold impairment correlates with hemoglobin reduction tendency except for female anosmia, post hoc one-way ANOVAs * *p* < 0.05.

Olfactory threshold wakening correlates with hemoglobin level alteration. MANOVA return significant differences in both males and females *p* < 0.05, F_(3,72)_ = 3.18 and F_(3,25)_ = 5.89, respectively. Post hoc one-way ANOVAs show significant differences *p* < 0.05 between normosmia vs. anosmia (F_(1,9)_ = 2.94), hyposmia vs. anosmia (F_(1,28)_ = 6.55), severe hyposmia vs. anosmia (F_(1,49)_ = 7.36) in males; and in females, normosmia vs. hyposmia (F_(1,9)_ = 31.51), severe hyposmia (F_(1,14)_ = 9.19) and anosmia (F_(1,8)_ = 19.43) and between hyposmia vs. anosmia (F_(1,10)_ = 13.07), severe hyposmia vs. anosmia (F_(1,15)_ = 4.98), (Figure 4).

### 3.5. Olfactory Threshold vs. HR

**Figure 5:**
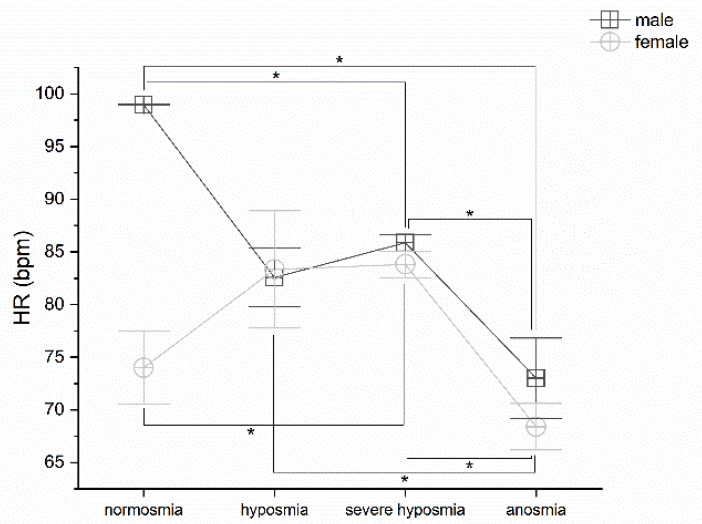
HR vs. olfactory threshold score in males and females. In both sexes, threshold impairment inversely correlates with sexes HR, post hoc one-way ANOVAs * *p* < 0.05.

Olfactory threshold damage correlates with heart rate variation. MANOVA returns significant differences in both males and females *p* < 0.05, F_(3,72)_ = 7.13 and F_(3,25)_ = 5.59, respectively. Post hoc one-way ANOVAs show significant differences *p* < 0.05 between normosmia vs. severe hyposmia (F_(1,43)_ = 12.95), normosmia vs. anosmia (F_(1,9)_ = 10.56), severe hyposmia vs. anosmia (F_(1,49)_ = 28.41) in males; and in females, normosmia vs. severe hyposmia (F_(1,14)_ = 11.50), hyposmia vs. anosmia (F_(1,10)_ = 5.31), severe hyposmia vs. anosmia (F_(1,15)_ = 41.93), (Figure 5).

### 3.6. Olfactory Threshold vs. BP

**Figure 6:**
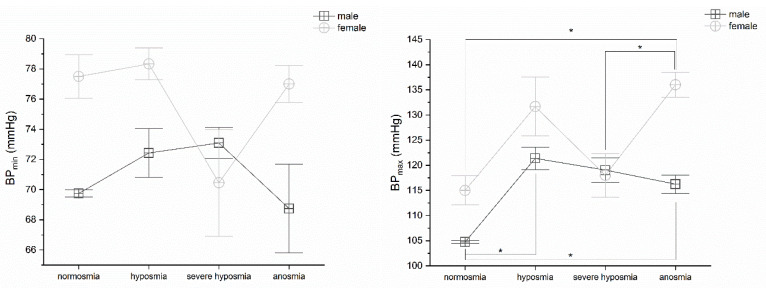
Blood pressure min and max values vs. olfactory threshold score in males and females. Significant differences occur only in BP_max_. For detail, see text, post hoc one-way ANOVAs * *p* < 0.05.

Olfactory threshold damage partially correlates with blood pressure alteration. MANOVA does not find any significant differences in BP_min_ in both sexes. On the contrary, BP_max_ MANOVA returns significant differences in females *p* < 0.05, F_(3,25)_ = 3.98. Post hoc one-way ANOVAs show significant differences *p* < 0.05 between normosmia vs. anosmia (F_(1,8)_ = 31.18) and severe hyposmia vs. anosmia (F_(1,15)_ = 7.18) in females. Nevertheless, in males, BP_max_ one-way ANOVA return significant differences in normosmia vs. hyposmia (F_(1,22)_ = 4.97) and anosmia (F_(1,9)_ = 9.02), (Figure 6).

## 4. Discussion

Among the most frequent causes of permanent loss of the sense of smell are acute upper respiratory tract infections induced by viruses, such as influenza viruses, rhinoviruses, coronaviruses, and respiratory syncytial viruses. Viruses produce partial lesions to the olfactory neuroepithelium; repeated damage from various infections can be cumulative and may lead to increased pathogenic vulnerability during ageing. Consequently, viruses, but also pollutants, may play a more important role than genetic predispositions in age-related olfactory impairment, as demonstrated by the non-linear decline in human olfactory phenotypes [40]. Olfactory sensory neurons regenerate throughout life after severe and repeated viral infections. This process leads to the degradation of the olfactory epithelium; it becomes thin and fragmented, with islands of metaplastic squamous epithelium containing few supporting cells and neurons with a reduced number of cilia [41]. SARS-CoV-2 can attack the peripheral olfactory system at the non-neuronal and neuronal cells. In the latter, for instance, it can interfere with the regeneration process [42]. Furthermore, in COVID-19 patients, radiological analysis of the morphology of the olfactory cleft points to the prevalence of opacification, suggesting impairment of the conductive mechanism [43]. The virus, therefore, appears to exert its damaging effect on multiple structures of the olfactory system [44].

In a previous report, we demonstrate the objective effects of SARS-CoV-2 on the olfactory threshold of COVID-19 patients by using a quick objective test [25], including asymptomatic patients [45]. As confirmed by other questionnaire-based studies [46], the rate of impairment of smell and ‘taste’ is broadly defined and difficult for people to report [47,48]. These works correctly explain that the results may be related to the sum of the lack of actual taste with the cross-modal perception of taste based on the combination of gustatory and olfactory stimuli [46,47,48]. Consequently, when the chemical senses are tested objectively, the result is a different picture, compared to self-assessment or questionnaires [25,49]. Consequently, testing the chemical senses objectively, the result shows a different picture, compared to self-assessment or questionnaires [25,49]. Due to the novelty of the effects of SARS-CoV-2 on olfaction, to understand both the mechanisms of viral aggression and its long-term consequences, it is crucial to investigate the potential cross-links between olfactory impairment and other physiological parameters. Several studies have attempted to associate olfactory impairment with various parameters from hospitalization, demographics, symptoms, degree of severity, comorbidities, and blood biomarkers while rarely being examined in correlation with physiological parameters [45]. Thus, in this paper, we correlate the results of the olfactory threshold test with general physiological parameters and the more specific hypoxia parameters to assess the possible cross-correlation between the neurological impairment of the sense of smell and the physiological imbalance due to the consequences of hypoxia.

The correlation between the deterioration of the olfactory threshold and alterations in physiological parameters and gender will be discussed in the following sections. 

### 4.1. Olfactory Threshold, Breath Frequency, pO_2_, pCO_2_, and Hemoglobin Levels

The study of the olfactory threshold is particularly suitable for investigating viral aggression. Recent work showed viral presence in the non-neural cells of the olfactory system and passage to the neural cells [42], probably by the mechanism of cellular linkage [50] is not excluded. Consequently, from the periphery, the olfactory pathway could allow the virus to enter the CNS through the cribriform plate, linking respiratory distress to viral aggression [51]. SARS-CoV-2 infection could attack and shut down the respiratory center of the CNS, which is potentially fatal because it blocks the work of the lungs [52]. SARS-CoV-2, as described for other viral diseases [51], could affect deeper parts of the brain, including the thalamus and brainstem, through trans-synaptic transfer, leading to the more complex pathogenesis of respiratory distress. COVID-19 can result in profound hypoxemia with near-normal arterial carbon dioxide (PaCO_2_) levels due to the improper distribution and shunt of the ventilation/perfusion ratio (VA/Q) and increased VE to increase CO_2_ removal from the lungs [52]. Hypoxemia from COVID-19 can cause dyspnea, defined as respiratory distress, or silent hypoxemia, or ‘happy’, which occurs when ventilation sensed through stretch receptors fails to meet demand conveyed by the corollary discharge from the brainstem to the cerebral cortex and paradoxically lung compliance (elasticity) still appears normal [52,53]. Dyspnea generally does not occur with hypoxemia alone, mainly if PCO_2_ is normal or near normal, but a secondary stimulus is needed, such as the activation of lung afferent neurons and/or CO_2_ chemoreceptors [52,53]. Heterogeneous consolidation in the lungs of COVID-19 patients leads to hypoxemia with normal or low PaCO_2_ [52,53]. A series of related events disrupt VA/Q relationships, resulting in increased respiratory mechanics and impaired gas exchange. Compromised VA/Q ratios cause blood gas values to become abnormal by activating central and peripheral chemoreceptors. Hypercapnic and hypoxic ventilatory responses and arterial chemoreflex function are complex tasks based on continuous CO_2_ assessment [52,53]. 

In agreement with this interpretation, we found that a change in breathing frequency in women correlates strongly with a reduction in the olfactory threshold, particularly in the case of severe hyposmia. This physiological reaction has not been observed in men, perhaps due to the hypothesized greater sensitivity to the virus in males. However, the olfactory threshold correlates with breathing frequency. Hyperventilation is the immediate acute response that compensates for the emerging hypoxia at high altitude, as occurs with the effect of the virus [34]. The silent hypoxemia observed in COVID-19 could be related to decreased erythrocyte counts [54,55] and low hematocrit [13]. These observations suggest that SARS-CoV-2 may act on multiple components of the respiratory system, leading to the alteration of oxygen uptake and transport and potentially also on the central regulation of respiration. In agreement, we observed a drastic decrease in pO_2_ related to olfactory threshold impairment; in fact, patients in normoxia show normal pO_2_ levels, whereas those with hyposmia, severe hyposmia, and anosmia have lower pO_2_ levels [52,53]. The pCO_2_ increases in both sexes in hyposmia and severe hyposmia. In anosmia, it increases in men and decreases in women [52]. The question remains, however, under which physiological conditions the virus spreads and contributes to pathogenesis.

Carotid body stimulation, which assesses CO_2_ and O_2_, is responsible for cardiovascular responses, due to increased sympathetic activity. Thus, in COVID-19 patients, the limited changes in heart rate during arterial desaturation may also indicate chemoreceptor impairment. Our results suggest CB involvement is due to the relationship between the hemoglobin O_2_ dissociation curve, arterial O_2_ content, and breath control [55]. Blood O_2_ capacity is determined mainly by hemoglobin concentration and saturation, with a limited contribution from dissolved O_2_. Within a few seconds, the CB responds to changes in arterial pO_2_. In pathological systemic hypoxia, physiological residents, and altitude visitors, pO_2_ may be altered. The CB chemo-transduction process in the various forms of hypoxia results, in hypoxic hypoxia, and in a low arterial pO_2_ associated with a decrease in arterial O_2_ content. This condition is probably due to low inspiratory pO_2_ at altitude or experimentally through alterations in inspired gas mixtures or inadequate alveolar ventilation in respiratory diseases and infections, such as COPD, sleep apnea, impaired lung diffusion, increased pulmonary shunt fraction [55], and putatively in COVID-19. In ischemic, histotoxic, and systemic hypoxia, CB induces hyperventilation; it is commonly accepted that the appropriate stimulus to CB is a fall in pO_2_ rather than O_2_ content or even HbO_2_ saturation. For this reason, a substantial change in the blood O_2_ capacity would be required to observe any effect on pO_2_ in the CB. In most but not all cases, the afferent discharge frequency of the CB remains unchanged with decreasing O_2_ content, induced, for example, by hemodilution [52,55].

This phenomenon has no effect on ventilation and may even reduce it, although the lack of ventilatory response could reflect a CNS-induced depression [52,55], which could be SARS-CoV-2. Alterations in pCO_2_ and pH in vivo can influence O_2_ administration through shifts in the position of the O_2_-haemoglobin dissociation curve; the interaction of these three stimuli must also be considered when interpreting data generated in vivo [52,55].

Similarly, the olfactory alteration induced by COVID-19 may indicate the virus’s effects on the peripheral and/or central nervous system. Indeed, olfactory-derived signals related to breathing that project to the hippocampus, prefrontal cortex, etc., are likely to be affected and, consequently, modify the sensation of dyspnea [52,53,55]. Possibly understanding the olfactory alteration, a hallmark of COVID-19, and the association with other phenotypes could disclose the underlying physiological mechanisms. Therefore, in COVID-19 patients, olfactory alteration analysis in hypoxemia and hypoxia may be very useful. Hemoglobin was below the normal range in most COVID-19 patients, especially in severe patients [54]. This finding agrees with our results. The explanation could be related to the inflammatory changes caused by the infection, which could interfere with erythropoiesis, resulting in reduced hemoglobin. The low incidence of anemia in COVID-19 may well be related to the long-life span of erythrocytes and their compensatory proliferation induced by the hypoxia associated with pneumonia [13]. No clinical differences exist among patients with haemoglobinopathy, compared to the general population. However, mortality due to COVID-19 is higher among patients with hemoglobinopathy [54].

In summary, COVID-19 unleashes a perfect storm in the respiratory system, like AMS, affecting the integrative layers of the respiratory system, damaging the lungs, impairing oxygen transport, compromising gas exchange and affecting the neural circuits that control breathing. Decreased olfactory threshold, combined with pO_2_, hemoglobin levels, increased respiratory rate, and pCO_2_, could be considered an indicator of disease severity and progression.

### 4.2. Olfactory Threshold vs. HR and BP

The infection probably alters the physiological balance between ACE and ACE2, resulting in RAS activation, abnormal vascular endothelial cell functions, and the coagulation system. The typical chronic disease is accompanied by severe cardiovascular and cerebrovascular disorders associated with vascular endothelial dysfunction [54]. D-dimer elevation and prothrombin time prolongation have been observed in severe cases of COVID-19 [54]. However, in severe infection, abnormal troponin measurements vary, and an elevated troponin in COVID-19 may not necessarily imply the occurrence of acute myocardial infarction or myocarditis [56]. We found heart rate changes correlated with impaired olfactory function and only a slight change in BPmax. Thus, heart failure and changes in blood pressure do not appear to be a direct effect of the virus. In contrast, they are more consistent with, for instance, its induced inflammation.

Further, chronic hypoxia increases AT1R expression and its efferent-mediated activity [30]. Moreover, AT1R activation increases intracellular calcium levels in dissociated glomus cells, an effect that is tripled by chronic hypoxia [57]. Hypoxia, through increased AT1R expression in the carotid chemoreceptor could influence changes in cardiopulmonary function.

Gender seems to play a role in the impairment of the sense of smell to the disadvantage of men [58]. AMS shows a similar dichotomy in the severity of the disease, showing sexual dimorphism with a more significant impact in males [37]. Accordingly, we found that for some parameters, gender significant differences, such as pCO_2_ and hemoglobin, further slight punctiform differences, e.g., in BF severe hyposmia. Punctiform dissimilarities could be linked to a series of prejudices. In contrast, structured discrepancies could conversely be attributable to certain physiological dimorphisms between the sexes. 

Based on our experimental results, it is possible to elaborate on at least two hypotheses: *i*. the direct effects on the respiratory center of the CNS by the viral aggression; *ii*. the indirect result of the infection producing hypoxia detected by the chemoreceptors in the carotid body. 

The direct central effect of the virus penetration via the olfactory system has been hypothesized and observed in similar virosis [51]. As first-order neurons, olfactory sensory neurons can directly transport xenobiotics from the environment to the brain. Loss of the sense of smell has been associated with early mortality and may signal the early stages of neurodegenerative disease [59]. The longevity of olfactory dysfunction associated with SARS-CoV-2 may increase predisposition to future neurological disorders [44,51]. Indeed, there is precedent to support this view. It is well known that after the 1918 influenza pandemic, referred to as the Spanish flu, about 80 per cent of individuals who recovered from lethargic encephalitis developed Parkinson’s-like symptoms [60]. By the hematogenous route or through the olfactory system, by direct infection of neurons or more complex indirect mechanisms, viral RNA reaches the leptomeningeal layers and the cerebral cortex causing olfactory dysfunction by disrupting signal propagation [42]. SARS-CoV-2 can spread in vitro from Vero E6 cells, the epithelial cell line infected by SARS-CoV-2, to SH-SY5Y cells lacking ACE2, the neuronal cell line not permissive to the virus, by using thin membrane ducts, TNTs, which form between infected and uninfected cells [50]. This evidence seems to point to an aetiology related to peripheral damage of the olfactory epithelium or its signal pathway, which would justify using an olfactory threshold test. For instance, potential viral targets in the central nervous system are two groups of neuronal networks crucial for the generation of respiratory rhythm: the pre-Bötzinger complex (PBC) and the retro-trapezoidal/parafacial respiratory group (RTN/pFRG) [27]. Lesions of the PBC cause lethality due to respiratory failure, even in the absence of other symptoms [28,29]. 

On the other hand, we may suppose that an indirect peripheral effect of viral aggression could influence Ang II functions locally in tissues and organs. This phenomenon is of interest for physiological and pathophysiological aspects. In CB, the existence of a system where key components of RAS, including ACE, are expressed in the absence of renin has been proposed, suggesting the existence of a renin-independent biosynthetic pathway for Ang II or an intrinsic angiotensin-generating system [30]. This mechanism could play a key role in modulating the CB response to hypoxia. Hypoxia regulates several critical components of the RAS in the CB, including time-dependent ACE activity [61]. 

The olfactory system is a sort of ‘viral sensor’, alerting of the pathogen’s presence. It is helpful for early diagnosis, which can be essential in interrupting the transmission of the virus. Available diagnostic techniques, e.g., radiographic and histological, cannot definitively rule out the trans-cribriform, transcellular, or paracellular passage of virions or subviral elements from the infected olfactory epithelium to the olfactory bulbs and into the CNS in patients with acute post-viral olfactory dysfunction. Immune-mediated olfactory neuropathy and encephalitic damage to the olfactory system are consistent with residual olfactory dysfunction with or without perceptual distortion, parosmia, and phantosmia [62,63].

Several studies suggest an association between chronic olfactory impairment and neurological effects [64,65]. Among the most consistent evidence is neuroinflammation within the brain. Indeed, olfactory brain areas are connected to adjacent brain regions involved in memory and attention and are deficient in the early stages of neurodegenerative diseases. The emerging hypothesis is that a chronic olfactory deficit in persons cured of COVID-19 may predict an increased likelihood of neurological sequelae or long-term neurodegenerative disorders. Long-term neurodegenerative sequelae may take years to manifest and may be clinically silent during the COVID-19 pandemic. Interestingly, the inflammation in the nasal epithelium induced by SARS-CoV-2 and in groups of patients with dementia are superimposable. It is conceivable that an inflammatory process from the nasal epithelium to the olfactory bulbs and related brain regions could, therefore, accelerate the pathological processes, leading to neurodegenerative disease [66]. 

A substantial percentage of COVID-19 patients may develop a long-lasting impairment of the sense of smell that could affect the predictive value of long COVID [67]. The prognostic value of olfactory impairment has been controversial in the panorama of published papers [65,67,68]. However, the controversy refers to different parameters, which are difficult to compare and evaluate in terms of the COVID-19 sequela. In this paper, we try to overcome such bias by coupling the olfactory threshold, considered with olfactometric methodology, to physiological parameters. Our results show a general correlation between threshold and physiological data, besides the gender variable introducing a degree of dissimilar changes with the worsening of olfactory functions. In particular, the most important difference between sexes in PCO_2_ and Hb. Other slight differences occur for severe hyposmia in BF and anosmia in BP. These results, however, have some limitations: Firstly, as in many other studies, the variant and sub-variant of SARS-CoV-2 could be a bias. Secondly, the previous effects of other rhinoviruses, influenza, parainfluenza, and Epstein–Barr virus were unknown. Thirdly, the patient’s basal parameters pre-COVID-19 are unknown. According to current knowledge, the pathogenesis of COVID-19 appears heterogeneous. It requires further studies on its pathophysiology through various systems’ homeostatic/functional interconnection. In agreement, we suggest that patients with COVID-19 undergo olfactory threshold screening in correlation with other physiological parameters. Currently, the brain is not considered a primary or secondary cause of death from infection. The cerebrospinal fluid of patients at different times of infection and the post-mortem brain tissue of COVID-19, like the functioning of the CB, was complex to analyze. However, if attention is paid to the impairment of the sense of smell, this difficulty could easily be overcome with the evaluation of the olfactory threshold, which could become the electrophysiological marker of brain engagement in disease.

## 5. Conclusions

In conclusion, these results show that a high percentage of COVID-19 patients have an impaired sense of smell, with the severity of the olfactory threshold impairment being related to alterations in physiological parameters. The prognostic role of chemosensory disorders assumes an increasing value because it correlates with a neurological phenomenon and with a broader homeostatic picture by correlating with some physiological parameters.

## Data Availability

All data information is in the paper and preserved in the repository of ENT unit of the ‘Ss. Annunziata’ University Hospital.

## References

[1] Sanche S., Lin Y.T., Xu C., Romero-Severson E., Hengartner N., Ke R. (2020). High Contagiousness and Rapid Spread of Severe Acute Respiratory Syndrome Coronavirus 2. Emerg. Infect. Dis..

[2] Huang C., Wang Y., Li X., Ren L., Zhao J., Hu Y., Zhang L., Fan G., Xu J., Gu X. (2020). Clinical features of patients infected with 2019 novel coronavirus in Wuhan, China. Lancet.

[3] Lovato A., de Filippis C. (2020). Clinical Presentation of COVID-19: A Systematic Review Focusing on Upper Airway Symptoms. Ear Nose Throat J..

[4] WHO WHO Director-General’s Remarks at the Media Briefing on 2019-nCoV on 11 February 2020. https://www.who.int/dg/speeches/detail/who-director-general-s-remarksat-the-media-briefing-on-2019-ncov-on-11-february-2020.

[5] Zhou P., Yang X.L., Wang X.G., Hu B., Zhang L., Zhang W., Si H.R., Zhu Y., Li B., Huang C.L. (2020). A pneumonia outbreak associated with a new coronavirus of probable bat origin. Nature.

[6] Hoffmann M., Kleine-Weber H., Schroeder S., Krüger N., Herrler T., Erichsen S., Schiergens T.S., Herrler G., Wu N.H., Nitsche A. (2020). SARS-CoV-2 Cell Entry Depends on ACE2 and TMPRSS2 and Is Blocked by a Clinically Proven Protease Inhibitor. Cell.

[7] Matsuyama S., Nagata N., Shirato K., Kawase M., Takeda M., Taguchi F. (2010). Efficient activation of the severe acute respiratory syndrome coronavirus spike protein by the transmembrane protease TMPRSS2. J. Virol..

[8] Glowacka I., Bertram S., Muller M.A., Allen P., Soilleux E., Pfefferle S., Steffen I., Tsegaye T.S., He Y., Gnirss K. (2011). Evidence that TMPRSS2 activates the severe acute respiratory syndrome coronavirus spike protein for membrane fusion and reduces viral control by the humoral immune response. J. Virol..

[9] Li Q., Guan X., Wu P., Wang X., Zhou L., Tong Y., Ren R., Leung K.S.M., Lau E.H.Y., Wong J.Y. (2020). Early Transmission Dynamics in Wuhan, China, of Novel Coronavirus-Infected Pneumonia. N. Engl. J. Med..

[10] Uhlén M., Fagerberg L., Hallström B.M., Lindskog C., Oksvold P., Mardinoglu A., Sivertsson Å., Kampf C., Sjöstedt E., Asplund A. (2015). Proteomics. Tissue-based map of the human proteome. Science.

[11] Sungnak W., Huang N., Bécavin C., Berg M., HCA Lung Biological Network (2020). SARS-CoV-2 Entry Genes Are Most Highly Expressed in Nasal Goblet and Ciliated Cells within Human Airways. Nat. Med..

[12] Zhou F., Yu T., Du R., Fan G., Liu Y., Liu Z., Xiang J., Wang Y., Song B., Gu X. (2020). Clinical course and risk factors for mortality of adult inpatients with COVID-19 in Wuhan, China: A retrospective cohort study. Lancet.

[13] Chalmers S., Khawaja A., Wieruszewski P.M., Gajic O., Odeyemi Y. (2019). Diagnosis and treatment of acute pulmonary inflammation in critically ill patients: The role of inflammatory biomarkers. World J. Crit. Care Med..

[14] Liu X., Zhang R., He G. (2020). Hematological findings in coronavirus disease 2019: Indications of progression of disease. Ann. Hematol..

[15] Reynolds H.R., Adhikari S., Pulgarin C., Troxel A.B., Iturrate E., Johnson S.B., Hausvater A., Newman J.D., Berger J.S., Bangalore S. (2020). Renin-Angiotensin-Aldosterone System Inhibitors and Risk of COVID-19. N. Engl. J. Med..

[16] Mao L., Jin H., Wang M., Hu Y., Chen S., He Q., Chang J., Hong C., Zhou Y., Wang D. (2020). Neurologic Manifestations of Hospitalized Patients With Coronavirus Disease 2019 in Wuhan, China. JAMA Neurol..

[17] Koralnik I.J., Tyler K.L. (2020). COVID-19: A Global Threat to the Nervous System. Ann. Neurol..

[18] Arabi Y.M., Harthi A., Hussein J., Bouchama A., Johani S., Hajeer A.H., Saeed B.T., Wahbi A., Saedy A., AlDabbagh T. (2015). Severe neurologic syndrome associated with Middle East respiratory syndrome corona virus (MERS-CoV). Infection.

[19] Kim J.E., Heo J.H., Kim H.O., Song S.H., Park S.S., Park T.H., Ahn J.Y., Kim M.K., Choi J.P. (2017). Neurological Complications during Treatment of Middle East Respiratory Syndrome. J. Clin. Neurol..

[20] Lau K.K., Yu W.C., Chu C.M., Lau S.T., Sheng B., Yuen K.Y. (2004). Possible central nervous system infection by SARS coronavirus. Emerg. Infect. Dis..

[21] Tsai L.K., Hsieh S.T., Chao C.C., Chen Y.C., Lin Y.H., Chang S.C., Chang Y.C. (2004). Neuromuscular disorders in severe acute respiratory syndrome. Arch. Neurol..

[22] Xu J., Zhong S., Liu J., Li L., Li Y., Wu X., Li Z., Deng P., Zhang J., Zhong N. (2005). Detection of severe acute respiratory syndrome coronavirus in the brain: Potential role of the chemokine mig in pathogenesis. Clin. Infect. Dis..

[23] Fotuhi M., Mianc A., Meysamid S., Rajic C.A. (2020). Neurobiology of COVID-19. J. Alzh. Dis..

[24] Hopkins C., Surda P., Kumar N. (2020). Presentation of new onset anosmia during the COVID-19 pandemic. Rhinology.

[25] Xydakis M.S., Dehgani-Mobaraki P., Holbrook E.H., Geisthoff U.W., Bauer C., Hautefort C., Herman P., Manley G.T., Lyon D.M., Hopkins C. (2020). Smell and taste dysfunction in patients with COVID-19. Lancet Infect. Dis..

[26] Mazzatenta A., Neri G., D’Ardes D., De Luca C., Marinari S., Porreca E., Cipollone F., Vecchiet J., Falcicchia C., Panichi V. (2020). Smell and Taste in Severe COVID-19: Self-Reported vs. Testing. Front. Med..

[27] Li Y.C., Bai W.Z., Hashikawa T. (2020). The neuroinvasive potential of SARS-CoV2 may play a role in the respiratory failure of COVID-19 patients. J. Med. Virol..

[28] Smith J., Ellenberger H., Ballanyi K., Richter D., Feldman J. (1991). PreBötzinger complex: A brainstem region that may generate respiratory rhythm in mammals. Science.

[29] Burgold T., Voituron N., Caganova M., Tripathi P.P., Menuet C., Tusi B.K., Spreafico F., Bévengut M., Gestreau C., Buontempo S. (2012). The H3K27 demethylase JMJD3 is required for maintenance of the embryonic respiratory neuronal network, neonatal breathing, and survival. Cell Rep..

[30] Whitcroft K.L., Hummel T. (2020). Olfactory Dysfunction in COVID-19: Diagnosis and Management. JAMA.

[31] Leung P.S. (2006). Novel roles of a local angiotensin-generating system in the carotid body. J. Physiol..

[32] Gonzalez C., Almaraz L., Obeso A., Rigual R. (1994). Carotid body chemoreceptors: From natural stimuli to sensory discharges. Physiol. Rev..

[33] Patel K.P., Schultz H.D. (2013). Angiotensin peptides and nitric oxide in 479 cardiovascular disease. Antioxid Redox Signal.

[34] Porzionato A., Emmi A., Stocco E., Barbon S., Boscolo-Berto R., Macchi V., De Caro R. (2020). The potential role of the Carotid Body in COVID-19. Am. J. Physiol. Lung. Cell. Mol. Physiol..

[35] Soliz J., Schneider-Gasser E.M., Arias-Reyes C., Aliaga-Raduan F., Poma-Machicao L., Zubieta-Callejac G., Furuya W.I., Trevizan-Baú E., Dhingra R.R., Dutschmann M. (2020). Coping with hypoxemia: Could erythropoietin (EPO) be an adjuvant treatment of COVID-19?. Resp. Physiol. Neurobiol..

[36] Gasmi A., Noor S., Tippairote T., Dadar M., Menzel A., Bjorklund G. (2020). Individual risk management strategy and potential therapeutic options for the COVID-19 pandemic. Clin. Immunol..

[37] Frisancho A.R. (1975). Functional adaptation to high altitude hypoxia. Science.

[38] Joseph V., Soliz J., Pequignot J., Sempore B., Cottet-Emard J.M., Dalmaz Y., Favier R., Spielvogel H., Pequignot J.M. (2000). Gender differentiation of the chemoreflex during growth at high altitude: Functional and neurochemical studies. Am. J. Physiol. Regul. Integr. Comp. Physiol..

[39] Basnyat B., Murdoch D.R. (2003). High-altitude illness. Lancet.

[40] Ruffini R., Di Giulio C., Verratti V., Pokorski M., Fanò-Illic G., Mazzatenta A. (2015). Adaptation of olfactory threshold at high altitude. Adv. Exp. Med. Biol..

[41] Mazzatenta A., Cellerino A., Origlia N., Barloscio D., Sartucci F., Di Giulio C., Domenici L. (2016). Olfactory phenotypic expression unveils human aging. Oncotarget.

[42] Yamagishi M., Hasegawa S., Nakano Y. (1988). Examination and classification of human olfactory mucosa in patients with clinical olfactory disturbances. Arch. Otorhinolaryngol..

[43] Khan M., Yoo S.J., Clijsters M., Backaert W., Vanstapel A., Speleman K., Van Gerven L. (2021). Visualizing in deceased COVID-19 patients how SARS-CoV-2 attacks the respiratory and olfactory mucosae but spares the olfactory bulb. Cell.

[44] Tan C.J., Tan B.K.J., Tan X.Y., Liu H.T., Teo C.B., See A., Xu S., Toh S.T., Kheok S.W., Charn T.C. (2022). Neuroradiological Basis of COVID-19 Olfactory Dysfunction: A Systematic Review and Meta-Analysis. Laryngoscope.

[45] Xydakis M.S., Albers M.W., Holbrook E.H., Lyon D.M., Shih R.Y., Frasnelli J.A., Pagenstecher A., Kupke A., Enquist L.W., Perlman S. (2021). Post-viral effects of COVID-19 in the olfactory system and their implications. Lancet Neurol..

[46] Mazzatenta A., Berardi A., Novarria G.A., Neri G. (2022). Unmasking the ‘Asymptomatic’ COVID-19: A Nose Question. Life.

[47] Izquierdo-Domínguez A., Rojas-Lechuga M.J., Chiesa-Estomba C., Calvo-Henríquez C., Ninchritz-Becerra E., Soriano-Reixach M., Poletti-Serafini D., Villarreal I.M., Maza-Solano J.M., Moreno-Luna R. (2020). Smell and taste dysfunction in COVID-19 is associated with younger age in ambulatory settings: A multicenter cross-sectional study. J. Investig. Allergol. Clin. Immunol..

[48] Hintschich C.A., Wenzel J.J., Hummel T., Hankir M.K., Kühnel T., Vielsmeier V., Bohr C. (2020). Psychophysical tests reveal impaired olfaction but preserved gustation in COVID-19 patients. Int. Forum. Allergy Rhinol..

[49] Escanilla O.D., Victor X.D., Di Lorenzo P.M. (2015). Odor-taste convergence in the nucleus of the solitary tract of the awake freely licking rat. J. Neurosci..

[50] Doty R.L., Smith R., McKeown D.A., Raj J. (1994). Tests of human olfactory function: Principal components analysis suggests that most measure a common source of variance. Percept. Psychophys..

[51] Pepe A., Pietropaoli S., Vos M., Barba-Spaeth G., Zurzolo C. (2022). Tunneling nanotubes provide a route for SARS-CoV-2 spreading. Sci. Adv..

[52] Dando S.J., Mackay-Sim A., Norton R., Currie B.J., St. John J.A., Ekberg J.A., Batzloff M., Ulett G.C., Beacham I.R. (2014). Pathogens penetrating the central nervous system: Infection pathways and the cellular and molecular mechanisms of invasion. Clin. Microbiol. Rev..

[53] Simonson T.S., Baker T.L., Banzett R.B., Bishop T., Dempsey J.A., Feldman J.L., Guyenet P.G., Hodson E.J., Mitchell G.S., Moya E.A. (2021). Silent hypoxaemia in COVID-19 patients. J. Physiol..

[54] Gattinoni L., Marini J.J., Camporota L. (2020). The Respiratory Drive: An Overlooked Tile of COVID-19 Pathophysiology. Am. J. Respir. Crit. Care Med..

[55] Chen N.S., Zhou M., Dong X., Qu J.M., Gong F.Y., Han Y., Qiu Y., Wang J., Liu Y., Wei Y. (2020). Epidemiological and clinical characteristics of 99 cases of 2019 novel coronavirus pneumonia in Wuhan, China: A descriptive study. Lancet.

[56] Kumar P., Prabhakar N.R. (2012). Peripheral chemoreceptors: Function and plasticity of the carotid body. Compr. Physiol..

[57] De Lorenzo A., Kasal D.A., Tura B.R., Lamas C.C., Rey H.C. (2020). Acute cardiac injury in patients with COVID-19. Am. J. Cardiovasc. Dis..

[58] Fung M.L., Lam S.Y., Dong X., Chen Y., Leung P.S. (2002). Postnatal hypoxemia increases angiotensin II sensitivity and up-regulates AT1a angiotensin receptors in rat carotid body chemoreceptors. J. Endocrinol..

[59] Moein S.T., Hashemian S.M., Tabarsi P., Doty R.L. (2020). Prevalence and reversibility of smell dysfunction measured psychophysically in a cohort of COVID-19 patients. Int. Forum. Allergy Rhinol..

[60] Doty R.L. (2017). Olfactory dysfunction in neurodegenerative diseases: Is there a common pathological substrate?. Lancet Neurol..

[61] Henry J., Smeyne R.J., Jang H., Miller B., Okun M.S. (2010). Parkinsonism and neurological mani-festations of influenza throughout the 20th and 21st centuries. Parkinsonism Relat. Disord..

[62] Lam S.Y., Fung M.L., Leung P.S. (2004). Regulation of the angiotensin-converting enzyme activity by a time-course hypoxia in the carotid body. J. Appl. Physiol..

[63] Netland J., Meyerholz D.K., Moore S., Cassell M., Perlman S. (2008). Severe acute respiratory syndrome coronavirus infection causes neuronal death in the absence of encephalitis in mice transgenic for human ACE2. J. Virol..

[64] Casez O., Willaume G., Grand S., Nemoz B., Lupo J., Kahane P., Brion J.P. (2021). Teaching NeuroImages: SARS-CoV-2-Related Encephalitis: MRI Pattern of Olfactory Tract Involvement. Neurology.

[65] Ismail I., Gad K. (2021). Absent blood oxygen level-dependent functional magnetic resonance imaging activation of the orbitofrontal cortex in a patient with persistent cacosmia and cacogeusia after COVID-19 infection. JAMA Neurol..

[66] Boscolo-Rizzo P., Menegaldo A., Fabbris C., Spinato G., Borsetto D., Vaira L.A., Calvanese L., Pettorelli A., Sonego M., Frezza D. (2021). Six-Month Psychophysical Evaluation of Olfactory Dysfunction in Patients with COVID-19. Chem. Senses.

[67] Xydakis M.S., Belluscio L. (2017). Detection of neurodegenerative disease using olfaction. Lancet Neurol..

[68] Tan B.K.J., Han R., Zhao J.J., Tan N.K.W., Quah E.S.H., Tan C.J., Chan Y.H., Teo N.W.Y., Charn T.C., See A. (2022). Prognosis and persistence of smell and taste dysfunction in patients with COVID-19: Meta-analysis with parametric cure modelling of recovery curves. BMJ.

